# Ethyl (1*R*,1′*S*,2′*S*,7a’*R*)-2-oxo-1′-[(3a*R*,5*R*,5a*S*,8a*S*,8b*R*)-2,2,7,7-tetra­methyl­tetra­hydro-3a*H*-bis­[1,3]dioxolo[4,5-*b*:4′,5′-*d*]pyran-5-yl]-1′,2′,5′,6′,7′,7a’-hexa­hydro-2*H*-spiro­[acenaphthyl­ene-1,3′-pyrrolizine]-2′-carboxyl­ate

**DOI:** 10.1107/S1600536811055760

**Published:** 2012-01-14

**Authors:** G. Jagadeesan, K. Sethusankar, R. Prasanna, R. Raghunathan

**Affiliations:** a Department of Physics, Dr MGR Educational and Research Institute, Dr MGR University, Chennai 600 095, India; b Department of Physics, RKM Vivekananda College (Autonomous), Chennai 600 004, India; c Department of Organic Chemistry, University of Madras, Maraimalai Campus, Chennai 600 025, India

## Abstract

In the title compound, C_32_H_37_NO_8_, the central pyran ring adopts a twist-boat conformation and the 1,3-dioxoane rings adopt envelope conformations. The acenaphthyl­enone unit and two C atoms of a pyrrolidine ring are disordered over two sets of sites [occupancy ratio 0.669 (7):0.331 (7)]. The major fraction of the disordered pyrrolidine ring exhibits an envelope conformation while the minor component is essentially planar [maximum deviation = 0.037 (12) Å]. The other pyrrolidine ring also adopts an envelope conformation. The dihedral angle between the mean planes of the two wings of the pyrrolidine ring is 30.6 (2)°. Both the major and minor components of the acenaphthyl­enone unit are essentially planar, the maximum deviations being 0.025 (10) and 0.047 (19) Å, respectively; the dihedral angle between the mean planes of the two components is 1.72 (3)°. The crystal packing features C—H⋯O inter­actions.

## Related literature

For applications of spiro­heterocycles, see: Ferguson *et al.* (2005 ▶). For a related structure, see: Athimoolam *et al.* (2008 ▶). For puckering parameters, see: Cremer & Pople (1975 ▶)
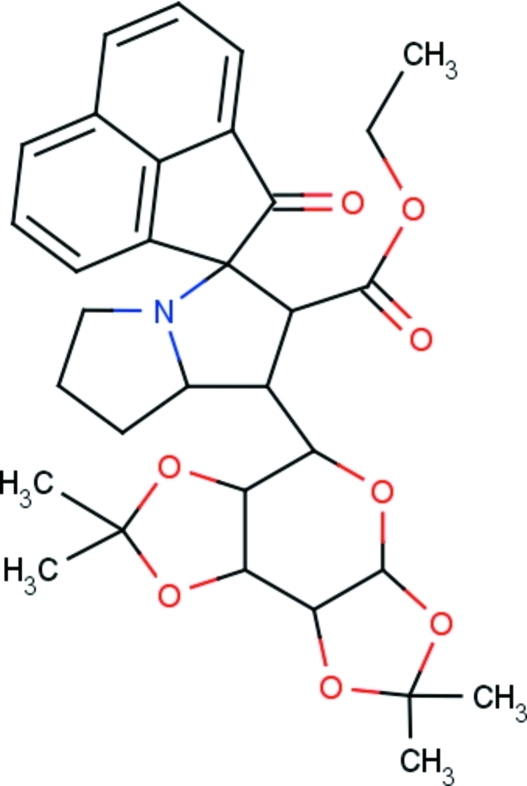


## Experimental

### 

#### Crystal data

C_32_H_37_NO_8_
*M* *_r_* = 563.63
Monoclinic, 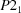

*a* = 11.4723 (4) Å
*b* = 8.9548 (2) Å
*c* = 15.0543 (5) Å
β = 96.990 (2)°
*V* = 1535.07 (8) Å^3^
*Z* = 2
Mo *K*α radiation
μ = 0.09 mm^−1^
*T* = 293 K
0.40 × 0.35 × 0.30 mm

#### Data collection

Bruker Kappa APEXII CCD diffractometer
Absorption correction: multi-scan (*SADABS*; Bruker, 2008 ▶) *T*_min_ = 0.966, *T*_max_ = 0.974
20295 measured reflections
5063 independent reflections
3469 reflections with *I* > 2σ(*I*)
*R*_int_ = 0.026

#### Refinement

*R*[*F* ^2^ > 2σ(*F* ^2^)] = 0.046
*wR*(*F* ^2^) = 0.124
*S* = 1.05
5039 reflections
413 parameters
50 restraints
H-atom parameters constrained
Δρ_max_ = 0.24 e Å^−3^
Δρ_min_ = −0.15 e Å^−3^

### 

Data collection: *APEX2* (Bruker, 2008 ▶); cell refinement: *SAINT* (Bruker, 2008 ▶); data reduction: *SAINT*; program(s) used to solve structure: *SHELXS97* (Sheldrick, 2008 ▶); program(s) used to refine structure: *SHELXL97* (Sheldrick, 2008 ▶); molecular graphics: *ORTEP-3* (Farrugia, 1997 ▶); software used to prepare material for publication: *SHELXL97* and *PLATON* (Spek, 2009 ▶).

## Supplementary Material

Crystal structure: contains datablock(s) global, I. DOI: 10.1107/S1600536811055760/pv2491sup1.cif

Structure factors: contains datablock(s) I. DOI: 10.1107/S1600536811055760/pv2491Isup2.hkl

Additional supplementary materials:  crystallographic information; 3D view; checkCIF report

## Figures and Tables

**Table 1:** Hydrogen-bond geometry (Å, °)

*D*—H⋯*A*	*D*—H	H⋯*A*	*D*⋯*A*	*D*—H⋯*A*
C4—H4⋯O8^i^	0.93	2.37	3.252 (9)	157
C26—H26⋯O1^ii^	0.98	2.38	3.211 (7)	143

Symmetry codes: (i) 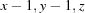; (ii) 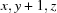.

**Fig. 1.**
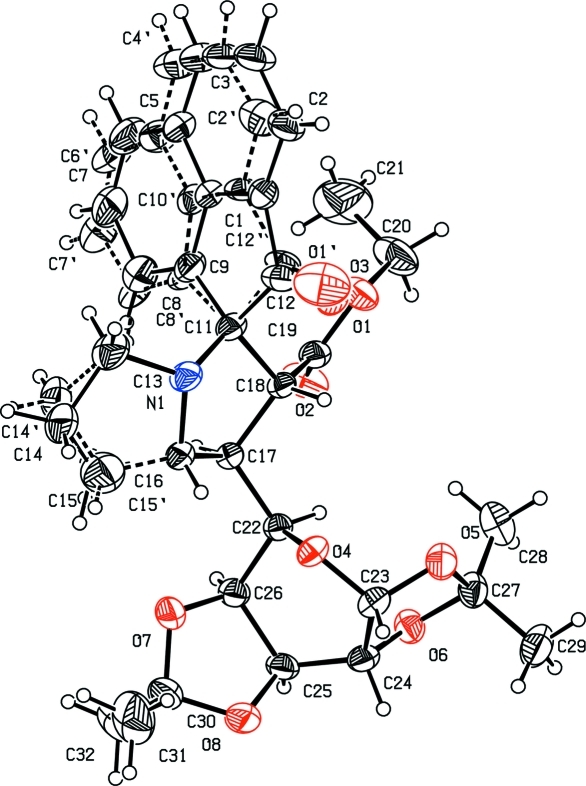
The molecular structure of the title compound with the atom numbering scheme, displacement ellipsoids are drawn at 30% probability level. H atoms are present as small spheres of arbitary radius. The minor fractions of the disordered acenapthenequinone and pyrrolidine groups have been represented by broken bonds.

**Fig. 2.**
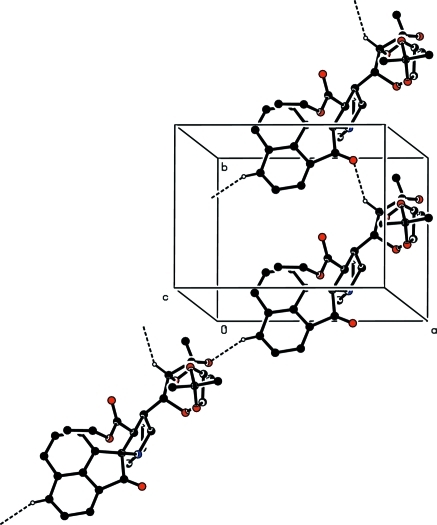
The packing arrangement of the tittle compound in the unit cell; The Dashed lines indicate the C–H···O interactions. H-atoms not involved in hydrogen bonds have been excluded for clarity.

## supplementary crystallographic information

### Comment

The design and novel synthesis of glycospiroheterocycles are interesting because of the synthetic challenges they present and their biological profile against viruses, bacteria, and cancer cells (Ferguson *et al.*, 2005). We have synthesized a novel glycospiroheterocyclic compound and determined its crystal structure which is presented in this article.

The tittle compound (Fig. 1) exhibits structural similarities with an already reported crystal structure of a closely related compound (Athimoolam *et al.*, 2008). The central pyran ring (C22–C26/O4) in the title compound adopts a *twist-boat* conformation with puckering parameters (Cremer & Pople, 1975): *Q* = 0.617 (2) Å, θ = 103.87 (19)° and φ = 220.2 (2)°. The 1,3–dioxoane rings, (C23/C24/O5/O6/C27) and (C25/C26/O7/O8/C30), adopt envelope conformations with O6 and C30 atoms 0.195 (2) and -0.200 (3) Å, respectively, out of the planes formed by the remaining atoms of the rings.

The acenaphthylenone moiety (C1–C12/O1) and C13 and C14 atoms of a pyrrolidine ring were disordered over two positions with site occupancy factors 0.670 (7):0.330 (7), representing major and minor components, respectively. The major fraction of the pyrrolidine ring N1/C13–C16 exhibits a C14-envelope conformation with C14 0.289 (9) Å out of the mean plane of the rest of the ring atoms while the minor component is essentially planar with maximum deviation of any atom from the mean plane formed by the rings atoms being 0.037 (12) Å for C13-atom. The pyrrolidine ring (N1/C11/C16—C18) adopts a C18-envelope conformation with C18 deviating from the mean plane of the remaining ring atoms by 0.225 (2) Å. The "butter-fly angle" between the mean planes N1/C11/C17/C18 and N1/C13/C15/C16 is 30.6 (2) °, in the pyrrolidine ring.

The major and minor components of the acenaphthylenone moiety (C1–C12/O1) are essentially planar with maximum deviations of atoms C2 and C1', 0.025 (10) Å and 0.047 (19) Å, respectively, and the dihedral angle between the mean planes of the two components is 1.72 (3)°. The crystal packing is stabilized by C—H···O hydrogen bonds *via* C4—H4···O8 and C26—H26···O1 intermolecular interactions (Tab. 1 and Fig. 2).

### Experimental

A mixture of 6,7-dideoxy-1,2:3,4-diisopropylidene-D-*galacto*-Oct- 6-enoethylpyranuronate (1 eq.), acenaphthenequinone (1 eq.) and *L*-proline (1.2 eq.) was refluxed at 413 K in toluene for about 6 hrs under Dean stark reaction condition to give 1,2:3,4-diisopropylidene-5-C[[2^'^.3^''^] spirooxindolo-3^'^-ethoxycarbonylpyrrolizidine]-*D*-galactopyranose. After the completion of reaction as indicated by TLC, solvent was evaporated under reduced pressure. The crude product was purified by column chromatography using hexane: EtOAc (4:1) as eluent. Block-shaped single crystals of the title compound suitable for X-ray diffraction analysis were obtained from a solution of hexane/ethyl acetate (4:1) by slow evaporation at room temperature.

### Refinement

The acenaphthylenone moiety (C1–C12/O1) and C13 and C14 atoms of the pyrrolidine ring were disordered over two positions with site occupancy factors 0.670 (7):0.330 (7). The bond lengths of both the major and minor components were restrainted to standard values using EADP and s.u. of 0.01 Å. The anisotropic displacement parameters of pairs of atoms in both components were restrained using ISOR with s.u. 0.01 using *SHELXL97* (Sheldrick, 2008). The hydrogen atoms were placed in calculated positions with C–H = 0.93 – 0.98 Å and refined in the riding mode with fixed isotropic displacement parameters: *U*_iso_(H) = 1.5 *U*_eq_(C) for methyl group and *U*_iso_(H) = 1.2 *U*_eq_(C) for other groups. In the absence of significant anomalous dispersion effects, an absolute structure was not determined, and 4797 Friedel pairs were merged.

### Crystal data

**Table:**

C_32_H_37_NO_8_	*F*(000) = 600
*M**_r_* = 563.63	*D*_x_ = 1.219 Mg m^−^^3^
Monoclinic, *P*2_1_	Mo *K*α radiation, λ = 0.71073 Å
Hall symbol: P 2yb	Cell parameters from 5063 reflections
*a* = 11.4723 (4) Å	θ = 1.4–30.7°
*b* = 8.9548 (2) Å	µ = 0.09 mm^−^^1^
*c* = 15.0543 (5) Å	*T* = 293 K
β = 96.990 (2)°	Block, colourless
*V* = 1535.07 (8) Å^3^	0.40 × 0.35 × 0.30 mm
*Z* = 2	

### Data collection

**Table:**

Bruker Kappa APEXII CCD diffractometer	5063 independent reflections
Radiation source: fine-focus sealed tube	3469 reflections with *I* > 2σ(*I*)
graphite	*R*_int_ = 0.026
ω scans	θ_max_ = 30.7°, θ_min_ = 2.4°
Absorption correction: multi-scan (*SADABS*; Bruker, 2008)	*h* = −15→16
*T*_min_ = 0.966, *T*_max_ = 0.974	*k* = −12→11
20295 measured reflections	*l* = −21→21

### Refinement

**Table:**

Refinement on *F*^2^	Primary atom site location: structure-invariant direct methods
Least-squares matrix: full	Secondary atom site location: difference Fourier map
*R*[*F*^2^ > 2σ(*F*^2^)] = 0.046	Hydrogen site location: inferred from neighbouring sites
*wR*(*F*^2^) = 0.124	H-atom parameters constrained
*S* = 1.05	*w* = 1/[σ^2^(*F*_o_^2^) + (0.0616*P*)^2^ + 0.0862*P*] where *P* = (*F*_o_^2^ + 2*F*_c_^2^)/3
5039 reflections	(Δ/σ)_max_ = 0.001
413 parameters	Δρ_max_ = 0.24 e Å^−^^3^
50 restraints	Δρ_min_ = −0.15 e Å^−^^3^

### Special details

**Table:**

Geometry. All e.s.d.'s (except the e.s.d. in the dihedral angle between two l.s. planes) are estimated using the full covariance matrix. The cell e.s.d.'s are taken into account individually in the estimation of e.s.d.'s in distances, angles and torsion angles; correlations between e.s.d.'s in cell parameters are only used when they are defined by crystal symmetry. An approximate (isotropic) treatment of cell e.s.d.'s is used for estimating e.s.d.'s involving l.s. planes.
Refinement. Refinement of *F*^2^ against ALL reflections. The weighted *R*-factor *wR* and goodness of fit *S* are based on *F*^2^, conventional *R*-factors *R* are based on *F*, with *F* set to zero for negative *F*^2^. The threshold expression of *F*^2^ > σ(*F*^2^) is used only for calculating *R*-factors(gt) *etc*. and is not relevant to the choice of reflections for refinement. *R*-factors based on *F*^2^ are statistically about twice as large as those based on *F*, and *R*- factors based on ALL data will be even larger.

### Fractional atomic coordinates and isotropic or equivalent isotropic displacement parameters (Å^2^)

**Table:**

	*x*	*y*	*z*	*U*_iso_*/*U*_eq_	Occ. (<1)
C11	0.59193 (19)	0.1469 (3)	0.22033 (14)	0.0479 (5)	
C1	0.4922 (6)	−0.0874 (7)	0.2582 (4)	0.0537 (15)	0.669 (7)
C2	0.4548 (9)	−0.2257 (11)	0.2815 (5)	0.083 (2)	0.669 (7)
H2	0.5074	−0.3002	0.3027	0.100*	0.669 (7)
C3	0.3315 (7)	−0.2493 (9)	0.2716 (5)	0.094 (2)	0.669 (7)
H3	0.3036	−0.3439	0.2832	0.113*	0.669 (7)
C4	0.2530 (7)	−0.1406 (11)	0.2462 (6)	0.098 (3)	0.669 (7)
H4	0.1731	−0.1620	0.2419	0.118*	0.669 (7)
C7	0.2673 (9)	0.2617 (13)	0.1818 (8)	0.092 (3)	0.669 (7)
H7	0.2190	0.3431	0.1656	0.111*	0.669 (7)
C5	0.2891 (6)	0.0050 (9)	0.2258 (4)	0.0734 (18)	0.669 (7)
C6	0.2182 (7)	0.1330 (12)	0.1992 (5)	0.085 (2)	0.669 (7)
H6	0.1368	0.1254	0.1943	0.102*	0.669 (7)
C8	0.3907 (15)	0.2805 (14)	0.1867 (10)	0.070 (2)	0.669 (7)
H8	0.4221	0.3728	0.1742	0.084*	0.669 (7)
C9	0.4635 (11)	0.1611 (11)	0.2100 (9)	0.0506 (18)	0.669 (7)
C10	0.4099 (7)	0.0250 (9)	0.2313 (5)	0.0528 (17)	0.669 (7)
C12	0.6119 (8)	−0.0220 (12)	0.2612 (8)	0.0480 (16)	0.669 (7)
O1	0.7037 (5)	−0.0726 (7)	0.2904 (4)	0.0670 (11)	0.669 (7)
C11'	0.59193 (19)	0.1469 (3)	0.22033 (14)	0.0479 (5)	0.00
C1'	0.4559 (16)	−0.0469 (19)	0.2460 (11)	0.0537 (15)	0.331 (7)
C2'	0.414 (2)	−0.193 (2)	0.2611 (12)	0.083 (2)	0.331 (7)
H2'	0.4620	−0.2746	0.2782	0.100*	0.331 (7)
C3'	0.285 (2)	−0.198 (3)	0.2462 (15)	0.094 (2)	0.331 (7)
H3'	0.2480	−0.2906	0.2471	0.113*	0.331 (7)
C4'	0.2176 (17)	−0.072 (2)	0.2311 (13)	0.098 (3)	0.331 (7)
H4'	0.1376	−0.0794	0.2351	0.118*	0.331 (7)
C5'	0.2616 (17)	0.063 (2)	0.2105 (11)	0.0734 (18)	0.331 (7)
C7'	0.268 (2)	0.324 (3)	0.1690 (19)	0.092 (3)	0.331 (7)
H7'	0.2281	0.4118	0.1522	0.111*	0.331 (7)
C6'	0.2076 (15)	0.207 (2)	0.1856 (11)	0.085 (2)	0.331 (7)
H6'	0.1263	0.2147	0.1814	0.102*	0.331 (7)
C8'	0.392 (3)	0.323 (4)	0.176 (2)	0.070 (2)	0.331 (7)
H8'	0.4331	0.4074	0.1612	0.084*	0.331 (7)
C9'	0.449 (3)	0.198 (3)	0.203 (2)	0.0506 (18)	0.331 (7)
C10'	0.3912 (17)	0.070 (2)	0.2172 (12)	0.0528 (17)	0.331 (7)
C12'	0.583 (2)	0.000 (3)	0.2520 (18)	0.0480 (16)	0.331 (7)
O1'	0.6700 (14)	−0.0789 (19)	0.2720 (11)	0.0670 (11)	0.331 (7)
C13	0.5891 (3)	0.1171 (5)	0.05317 (18)	0.0780 (9)	
H13A	0.5054	0.1335	0.0526	0.094*	0.669 (7)
H13B	0.6022	0.0135	0.0383	0.094*	0.669 (7)
C14	0.6416 (7)	0.2255 (11)	−0.0147 (4)	0.0864 (19)	0.669 (7)
H14A	0.7167	0.1905	−0.0299	0.104*	0.669 (7)
H14B	0.5879	0.2398	−0.0690	0.104*	0.669 (7)
C15	0.6546 (16)	0.368 (3)	0.0428 (13)	0.083 (3)	0.669 (7)
H15A	0.5791	0.4131	0.0490	0.100*	0.669 (7)
H15B	0.7049	0.4412	0.0189	0.100*	0.669 (7)
C13'	0.5891 (3)	0.1171 (5)	0.05317 (18)	0.0780 (9)	0.00
H13C	0.6234	0.0300	0.0283	0.094*	0.331 (7)
H13D	0.5071	0.0968	0.0583	0.094*	0.331 (7)
C14'	0.5997 (15)	0.238 (3)	0.0017 (10)	0.0864 (19)	0.331 (7)
H14C	0.5227	0.2809	−0.0153	0.104*	0.331 (7)
H14D	0.6308	0.2072	−0.0526	0.104*	0.331 (7)
C15'	0.682 (4)	0.358 (7)	0.050 (3)	0.083 (3)	0.331 (7)
H15C	0.7497	0.3742	0.0190	0.100*	0.331 (7)
H15D	0.6402	0.4518	0.0536	0.100*	0.331 (7)
C16	0.7142 (2)	0.2993 (3)	0.13480 (14)	0.0503 (5)	
H16	0.7981	0.2820	0.1320	0.060*	
C17	0.69672 (17)	0.3832 (3)	0.22092 (13)	0.0416 (4)	
H17	0.6296	0.4508	0.2080	0.050*	
C18	0.66266 (18)	0.2589 (2)	0.28307 (14)	0.0406 (4)	
H18	0.7348	0.2094	0.3095	0.049*	
C19	0.5980 (2)	0.3121 (3)	0.35780 (15)	0.0491 (5)	
C20	0.5076 (4)	0.2307 (5)	0.4838 (2)	0.0924 (11)	
H20A	0.5346	0.3249	0.5106	0.111*	
H20B	0.5229	0.1533	0.5288	0.111*	
C21	0.3815 (4)	0.2393 (9)	0.4553 (4)	0.146 (2)	
H21A	0.3411	0.2600	0.5061	0.220*	
H21B	0.3545	0.1458	0.4291	0.220*	
H21C	0.3660	0.3176	0.4119	0.220*	
C22	0.80032 (17)	0.4724 (2)	0.26659 (14)	0.0411 (4)	
H22	0.7875	0.4879	0.3291	0.049*	
C23	0.99820 (19)	0.4371 (3)	0.32680 (16)	0.0505 (5)	
H23	1.0723	0.4010	0.3085	0.061*	
C24	1.0038 (2)	0.6074 (3)	0.33687 (15)	0.0532 (6)	
H24	1.0859	0.6402	0.3423	0.064*	
C25	0.9321 (2)	0.6970 (3)	0.26499 (14)	0.0538 (6)	
H25	0.9173	0.7972	0.2872	0.065*	
C26	0.8158 (2)	0.6236 (3)	0.22544 (14)	0.0489 (5)	
H26	0.7493	0.6886	0.2338	0.059*	
C27	0.9899 (2)	0.5074 (3)	0.47405 (14)	0.0560 (6)	
C28	0.8969 (3)	0.4856 (4)	0.5344 (2)	0.0803 (9)	
H28A	0.9167	0.4013	0.5727	0.120*	
H28B	0.8228	0.4682	0.4990	0.120*	
H28C	0.8917	0.5734	0.5703	0.120*	
C29	1.1111 (3)	0.5247 (5)	0.5240 (2)	0.0831 (9)	
H29A	1.1300	0.4381	0.5606	0.125*	
H29B	1.1134	0.6119	0.5613	0.125*	
H29C	1.1672	0.5350	0.4820	0.125*	
C30	0.9167 (3)	0.7058 (4)	0.11239 (17)	0.0687 (7)	
C31	0.9768 (4)	0.6396 (7)	0.0394 (2)	0.1171 (16)	
H31A	1.0370	0.7063	0.0247	0.176*	
H31B	0.9206	0.6239	−0.0125	0.176*	
H31C	1.0114	0.5457	0.0590	0.176*	
C32	0.8670 (4)	0.8595 (4)	0.0899 (3)	0.1013 (12)	
H32A	0.9286	0.9243	0.0755	0.152*	
H32B	0.8335	0.8989	0.1404	0.152*	
H32C	0.8074	0.8527	0.0394	0.152*	
O2	0.5742 (2)	0.4391 (2)	0.37177 (15)	0.0771 (6)	
O3	0.57205 (18)	0.1979 (2)	0.40881 (11)	0.0679 (5)	
O4	0.90450 (12)	0.38520 (18)	0.26594 (10)	0.0474 (3)	
O5	0.98714 (16)	0.3842 (2)	0.41297 (11)	0.0607 (4)	
O6	0.95792 (15)	0.63306 (19)	0.41854 (10)	0.0552 (4)	
O7	0.82740 (17)	0.6087 (2)	0.13311 (10)	0.0651 (5)	
O8	0.99739 (17)	0.7064 (3)	0.19099 (12)	0.0802 (6)	
N1	0.65251 (17)	0.1553 (2)	0.14046 (12)	0.0539 (5)	

### Atomic displacement parameters (Å^2^)

**Table:**

	*U*^11^	*U*^22^	*U*^33^	*U*^12^	*U*^13^	*U*^23^
C11	0.0449 (11)	0.0493 (13)	0.0481 (11)	−0.0126 (9)	−0.0001 (9)	−0.0063 (9)
C1	0.070 (4)	0.046 (4)	0.045 (2)	−0.020 (2)	0.006 (2)	−0.005 (2)
C2	0.125 (8)	0.074 (5)	0.050 (4)	−0.044 (4)	0.009 (3)	0.002 (3)
C3	0.115 (5)	0.090 (4)	0.079 (4)	−0.065 (4)	0.020 (3)	0.003 (3)
C4	0.088 (6)	0.117 (7)	0.092 (4)	−0.061 (5)	0.022 (4)	−0.005 (5)
C7	0.0487 (18)	0.144 (9)	0.081 (4)	0.012 (5)	−0.006 (2)	0.017 (5)
C5	0.057 (3)	0.108 (5)	0.054 (3)	−0.028 (3)	0.000 (2)	0.003 (3)
C6	0.047 (2)	0.131 (6)	0.073 (3)	−0.012 (4)	−0.005 (2)	0.008 (4)
C8	0.0505 (15)	0.089 (8)	0.067 (4)	0.001 (6)	−0.007 (3)	0.015 (4)
C9	0.035 (4)	0.064 (6)	0.050 (2)	−0.002 (3)	−0.006 (2)	−0.002 (4)
C10	0.051 (3)	0.067 (6)	0.038 (3)	−0.021 (4)	−0.005 (2)	0.007 (3)
C12	0.054 (6)	0.038 (4)	0.049 (3)	0.000 (3)	−0.003 (3)	−0.005 (2)
O1	0.069 (4)	0.0460 (13)	0.078 (3)	0.000 (2)	−0.021 (2)	−0.0076 (18)
C11'	0.0449 (11)	0.0493 (13)	0.0481 (11)	−0.0126 (9)	−0.0001 (9)	−0.0063 (9)
C1'	0.070 (4)	0.046 (4)	0.045 (2)	−0.020 (2)	0.006 (2)	−0.005 (2)
C2'	0.125 (8)	0.074 (5)	0.050 (4)	−0.044 (4)	0.009 (3)	0.002 (3)
C3'	0.115 (5)	0.090 (4)	0.079 (4)	−0.065 (4)	0.020 (3)	0.003 (3)
C4'	0.088 (6)	0.117 (7)	0.092 (4)	−0.061 (5)	0.022 (4)	−0.005 (5)
C5'	0.057 (3)	0.108 (5)	0.054 (3)	−0.028 (3)	0.000 (2)	0.003 (3)
C7'	0.0487 (18)	0.144 (9)	0.081 (4)	0.012 (5)	−0.006 (2)	0.017 (5)
C6'	0.047 (2)	0.131 (6)	0.073 (3)	−0.012 (4)	−0.005 (2)	0.008 (4)
C8'	0.0505 (15)	0.089 (8)	0.067 (4)	0.001 (6)	−0.007 (3)	0.015 (4)
C9'	0.035 (4)	0.064 (6)	0.050 (2)	−0.002 (3)	−0.006 (2)	−0.002 (4)
C10'	0.051 (3)	0.067 (6)	0.038 (3)	−0.021 (4)	−0.005 (2)	0.007 (3)
C12'	0.054 (6)	0.038 (4)	0.049 (3)	0.000 (3)	−0.003 (3)	−0.005 (2)
O1'	0.069 (4)	0.0460 (13)	0.078 (3)	0.000 (2)	−0.021 (2)	−0.0076 (18)
C13	0.085 (2)	0.095 (2)	0.0534 (14)	−0.0276 (18)	0.0040 (13)	−0.0244 (15)
C14	0.092 (5)	0.131 (4)	0.036 (3)	−0.038 (5)	0.007 (2)	−0.009 (3)
C15	0.110 (8)	0.087 (5)	0.044 (4)	−0.026 (7)	−0.027 (5)	0.012 (3)
C13'	0.085 (2)	0.095 (2)	0.0534 (14)	−0.0276 (18)	0.0040 (13)	−0.0244 (15)
C14'	0.092 (5)	0.131 (4)	0.036 (3)	−0.038 (5)	0.007 (2)	−0.009 (3)
C15'	0.110 (8)	0.087 (5)	0.044 (4)	−0.026 (7)	−0.027 (5)	0.012 (3)
C16	0.0503 (12)	0.0582 (14)	0.0416 (11)	−0.0148 (11)	0.0014 (9)	−0.0044 (10)
C17	0.0389 (10)	0.0408 (10)	0.0438 (10)	−0.0055 (8)	0.0006 (8)	0.0021 (8)
C18	0.0383 (10)	0.0382 (10)	0.0444 (10)	−0.0076 (8)	0.0018 (8)	−0.0025 (8)
C19	0.0505 (12)	0.0476 (13)	0.0490 (11)	−0.0121 (10)	0.0060 (9)	−0.0022 (9)
C20	0.128 (3)	0.092 (3)	0.0654 (18)	−0.023 (2)	0.0465 (18)	−0.0008 (17)
C21	0.112 (3)	0.202 (7)	0.141 (4)	−0.018 (4)	0.075 (3)	−0.006 (4)
C22	0.0405 (10)	0.0402 (11)	0.0427 (10)	−0.0050 (8)	0.0053 (8)	−0.0008 (8)
C23	0.0423 (11)	0.0542 (13)	0.0544 (12)	−0.0048 (10)	0.0034 (9)	−0.0086 (10)
C24	0.0506 (12)	0.0584 (14)	0.0499 (12)	−0.0208 (11)	0.0028 (9)	−0.0062 (10)
C25	0.0671 (14)	0.0473 (12)	0.0481 (11)	−0.0229 (11)	0.0110 (10)	−0.0011 (10)
C26	0.0513 (12)	0.0451 (12)	0.0509 (11)	−0.0065 (10)	0.0082 (9)	0.0022 (9)
C27	0.0690 (15)	0.0509 (13)	0.0453 (11)	0.0007 (11)	−0.0041 (10)	−0.0015 (10)
C28	0.100 (2)	0.081 (2)	0.0606 (15)	0.0148 (18)	0.0147 (15)	0.0169 (15)
C29	0.082 (2)	0.086 (2)	0.0726 (18)	−0.0016 (17)	−0.0245 (15)	−0.0066 (16)
C30	0.0733 (17)	0.0831 (19)	0.0518 (13)	−0.0191 (15)	0.0159 (12)	0.0057 (13)
C31	0.124 (3)	0.166 (5)	0.0657 (18)	0.017 (3)	0.0325 (19)	−0.001 (3)
C32	0.144 (3)	0.080 (2)	0.085 (2)	−0.020 (2)	0.031 (2)	0.0277 (19)
O2	0.0996 (15)	0.0524 (11)	0.0876 (14)	−0.0034 (10)	0.0457 (12)	−0.0075 (10)
O3	0.0907 (13)	0.0615 (11)	0.0554 (9)	−0.0129 (10)	0.0244 (9)	0.0028 (8)
O4	0.0422 (8)	0.0447 (8)	0.0537 (8)	−0.0021 (6)	−0.0013 (6)	−0.0106 (7)
O5	0.0763 (11)	0.0476 (9)	0.0547 (10)	−0.0008 (9)	−0.0065 (8)	−0.0003 (7)
O6	0.0745 (11)	0.0457 (9)	0.0439 (8)	−0.0050 (8)	0.0012 (7)	−0.0021 (7)
O7	0.0823 (12)	0.0679 (11)	0.0429 (8)	−0.0218 (10)	−0.0013 (8)	0.0071 (8)
O8	0.0672 (11)	0.1192 (18)	0.0555 (10)	−0.0378 (12)	0.0127 (8)	0.0105 (11)
N1	0.0560 (11)	0.0599 (12)	0.0452 (9)	−0.0163 (9)	0.0041 (8)	−0.0123 (8)

### Geometric parameters (Å, °)

**Table:**

C11—N1	1.462 (3)	C15'—C16	1.39 (4)
C11—C9	1.468 (12)	C15'—H15C	0.9700
C11—C18	1.540 (3)	C15'—H15D	0.9700
C11—C12	1.638 (10)	C16—N1	1.479 (3)
C1—C2	1.370 (11)	C16—C17	1.533 (3)
C1—C10	1.406 (7)	C16—H16	0.9800
C1—C12	1.490 (14)	C17—C22	1.525 (3)
C2—C3	1.419 (13)	C17—C18	1.535 (3)
C2—H2	0.9300	C17—H17	0.9800
C3—C4	1.350 (11)	C18—C19	1.498 (3)
C3—H3	0.9300	C18—H18	0.9800
C4—C5	1.413 (11)	C19—O2	1.194 (3)
C4—H4	0.9300	C19—O3	1.334 (3)
C7—C6	1.324 (13)	C20—O3	1.453 (3)
C7—C8	1.42 (2)	C20—C21	1.460 (7)
C7—H7	0.9300	C20—H20A	0.9700
C5—C10	1.389 (10)	C20—H20B	0.9700
C5—C6	1.434 (11)	C21—H21A	0.9600
C6—H6	0.9300	C21—H21B	0.9600
C8—C9	1.376 (13)	C21—H21C	0.9600
C8—H8	0.9300	C22—O4	1.429 (3)
C9—C10	1.419 (15)	C22—C26	1.508 (3)
C12—O1	1.181 (8)	C22—H22	0.9800
C1'—C10'	1.329 (17)	C23—O5	1.401 (3)
C1'—C2'	1.42 (3)	C23—O4	1.404 (3)
C1'—C12'	1.51 (3)	C23—C24	1.534 (4)
C2'—C3'	1.47 (3)	C23—H23	0.9800
C2'—H2'	0.9300	C24—O6	1.414 (3)
C3'—C4'	1.38 (3)	C24—C25	1.508 (4)
C3'—H3'	0.9300	C24—H24	0.9800
C4'—C5'	1.36 (2)	C25—O8	1.418 (3)
C4'—H4'	0.9300	C25—C26	1.541 (3)
C5'—C6'	1.45 (2)	C25—H25	0.9800
C5'—C10'	1.48 (3)	C26—O7	1.419 (3)
C7'—C6'	1.30 (3)	C26—H26	0.9800
C7'—C8'	1.42 (5)	C27—O6	1.423 (3)
C7'—H7'	0.9300	C27—O5	1.434 (3)
C6'—H6'	0.9300	C27—C28	1.496 (4)
C8'—C9'	1.33 (3)	C27—C29	1.506 (4)
C8'—H8'	0.9300	C28—H28A	0.9600
C9'—C10'	1.35 (4)	C28—H28B	0.9600
C12'—O1'	1.23 (2)	C28—H28C	0.9600
C13—N1	1.463 (3)	C29—H29A	0.9600
C13—C14	1.582 (8)	C29—H29B	0.9600
C13—H13A	0.9700	C29—H29C	0.9600
C13—H13B	0.9700	C30—O7	1.408 (3)
C14—C15	1.54 (3)	C30—O8	1.411 (3)
C14—H14A	0.9700	C30—C31	1.489 (5)
C14—H14B	0.9700	C30—C32	1.512 (5)
C15—C16	1.592 (16)	C31—H31A	0.9600
C15—H15A	0.9700	C31—H31B	0.9600
C15—H15B	0.9700	C31—H31C	0.9600
C14'—C15'	1.55 (5)	C32—H32A	0.9600
C14'—H14C	0.9700	C32—H32B	0.9600
C14'—H14D	0.9700	C32—H32C	0.9600
			
N1—C11—C9	118.7 (5)	C15—C16—H16	110.1
N1—C11—C18	101.73 (16)	C22—C17—C16	117.59 (17)
C9—C11—C18	117.1 (5)	C22—C17—C18	110.33 (16)
N1—C11—C12	107.3 (5)	C16—C17—C18	103.26 (18)
C9—C11—C12	102.3 (5)	C22—C17—H17	108.4
C18—C11—C12	109.4 (4)	C16—C17—H17	108.4
C2—C1—C10	120.1 (7)	C18—C17—H17	108.4
C2—C1—C12	131.6 (6)	C19—C18—C17	114.40 (18)
C10—C1—C12	108.2 (6)	C19—C18—C11	113.31 (17)
C1—C2—C3	116.5 (7)	C17—C18—C11	104.64 (16)
C1—C2—H2	121.7	C19—C18—H18	108.1
C3—C2—H2	121.7	C17—C18—H18	108.1
C4—C3—C2	123.0 (6)	C11—C18—H18	108.1
C4—C3—H3	118.5	O2—C19—O3	123.8 (2)
C2—C3—H3	118.5	O2—C19—C18	125.4 (2)
C3—C4—C5	121.5 (6)	O3—C19—C18	110.7 (2)
C3—C4—H4	119.2	O3—C20—C21	111.2 (3)
C5—C4—H4	119.2	O3—C20—H20A	109.4
C6—C7—C8	122.6 (8)	C21—C20—H20A	109.4
C6—C7—H7	118.7	O3—C20—H20B	109.4
C8—C7—H7	118.7	C21—C20—H20B	109.4
C10—C5—C4	115.1 (7)	H20A—C20—H20B	108.0
C10—C5—C6	116.2 (6)	C20—C21—H21A	109.5
C4—C5—C6	128.8 (6)	C20—C21—H21B	109.5
C7—C6—C5	120.7 (7)	H21A—C21—H21B	109.5
C7—C6—H6	119.6	C20—C21—H21C	109.5
C5—C6—H6	119.6	H21A—C21—H21C	109.5
C9—C8—C7	119.5 (10)	H21B—C21—H21C	109.5
C9—C8—H8	120.3	O4—C22—C26	110.34 (16)
C7—C8—H8	120.3	O4—C22—C17	108.49 (17)
C8—C9—C10	117.3 (11)	C26—C22—C17	114.17 (18)
C8—C9—C11	131.6 (11)	O4—C22—H22	107.9
C10—C9—C11	111.0 (5)	C26—C22—H22	107.9
C5—C10—C1	123.7 (7)	C17—C22—H22	107.9
C5—C10—C9	123.7 (6)	O5—C23—O4	110.12 (18)
C1—C10—C9	112.7 (7)	O5—C23—C24	104.60 (19)
O1—C12—C1	129.6 (10)	O4—C23—C24	114.4 (2)
O1—C12—C11	124.9 (10)	O5—C23—H23	109.2
C1—C12—C11	105.4 (4)	O4—C23—H23	109.2
C10'—C1'—C2'	127 (2)	C24—C23—H23	109.2
C10'—C1'—C12'	107.3 (19)	O6—C24—C25	108.0 (2)
C2'—C1'—C12'	126.0 (18)	O6—C24—C23	103.40 (19)
C1'—C2'—C3'	110.9 (17)	C25—C24—C23	116.4 (2)
C1'—C2'—H2'	124.5	O6—C24—H24	109.6
C3'—C2'—H2'	124.5	C25—C24—H24	109.6
C4'—C3'—C2'	122.4 (19)	C23—C24—H24	109.6
C4'—C3'—H3'	118.8	O8—C25—C24	107.5 (2)
C2'—C3'—H3'	118.8	O8—C25—C26	103.71 (17)
C5'—C4'—C3'	123.5 (19)	C24—C25—C26	114.92 (19)
C5'—C4'—H4'	118.2	O8—C25—H25	110.1
C3'—C4'—H4'	118.2	C24—C25—H25	110.1
C4'—C5'—C6'	133.3 (19)	C26—C25—H25	110.1
C4'—C5'—C10'	114.8 (18)	O7—C26—C22	110.37 (19)
C6'—C5'—C10'	111.9 (15)	O7—C26—C25	103.79 (17)
C6'—C7'—C8'	123 (2)	C22—C26—C25	111.34 (19)
C6'—C7'—H7'	118.7	O7—C26—H26	110.4
C8'—C7'—H7'	118.7	C22—C26—H26	110.4
C7'—C6'—C5'	122.8 (17)	C25—C26—H26	110.4
C7'—C6'—H6'	118.6	O6—C27—O5	104.34 (15)
C5'—C6'—H6'	118.6	O6—C27—C28	107.9 (2)
C9'—C8'—C7'	118 (3)	O5—C27—C28	109.1 (2)
C9'—C8'—H8'	120.8	O6—C27—C29	111.6 (2)
C7'—C8'—H8'	120.9	O5—C27—C29	110.3 (2)
C8'—C9'—C10'	122 (3)	C28—C27—C29	113.2 (2)
C1'—C10'—C9'	117 (2)	C27—C28—H28A	109.5
C1'—C10'—C5'	120.3 (18)	C27—C28—H28B	109.5
C9'—C10'—C5'	122.1 (18)	H28A—C28—H28B	109.5
O1'—C12'—C1'	127 (3)	C27—C28—H28C	109.5
N1—C13—C14	104.5 (3)	H28A—C28—H28C	109.5
N1—C13—H13A	110.9	H28B—C28—H28C	109.5
C14—C13—H13A	110.9	C27—C29—H29A	109.5
N1—C13—H13B	110.9	C27—C29—H29B	109.5
C14—C13—H13B	110.9	H29A—C29—H29B	109.5
H13A—C13—H13B	108.9	C27—C29—H29C	109.5
C15—C14—C13	99.4 (10)	H29A—C29—H29C	109.5
C15—C14—H14A	111.9	H29B—C29—H29C	109.5
C13—C14—H14A	111.9	O7—C30—O8	103.7 (2)
C15—C14—H14B	111.9	O7—C30—C31	109.4 (3)
C13—C14—H14B	111.9	O8—C30—C31	107.8 (3)
H14A—C14—H14B	109.6	O7—C30—C32	110.3 (3)
C14—C15—C16	99.8 (14)	O8—C30—C32	112.1 (3)
C14—C15—H15A	111.8	C31—C30—C32	113.1 (3)
C16—C15—H15A	111.8	C30—C31—H31A	109.5
C14—C15—H15B	111.8	C30—C31—H31B	109.5
C16—C15—H15B	111.8	H31A—C31—H31B	109.5
H15A—C15—H15B	109.5	C30—C31—H31C	109.5
C15'—C14'—H14C	109.1	H31A—C31—H31C	109.5
C15'—C14'—H14D	109.1	H31B—C31—H31C	109.5
H14C—C14'—H14D	107.9	C30—C32—H32A	109.5
C16—C15'—C14'	104 (4)	C30—C32—H32B	109.5
C16—C15'—H15C	110.9	H32A—C32—H32B	109.5
C14'—C15'—H15C	110.9	C30—C32—H32C	109.5
C16—C15'—H15D	110.9	H32A—C32—H32C	109.5
C14'—C15'—H15D	110.9	H32B—C32—H32C	109.5
H15C—C15'—H15D	108.9	C19—O3—C20	117.6 (2)
C15'—C16—N1	108 (2)	C23—O4—C22	112.91 (17)
C15'—C16—C17	123 (2)	C23—O5—C27	109.65 (18)
N1—C16—C17	105.42 (17)	C24—O6—C27	106.59 (19)
N1—C16—C15	103.3 (9)	C30—O7—C26	108.47 (18)
C17—C16—C15	117.3 (9)	C30—O8—C25	107.68 (19)
C15'—C16—H16	100.2	C13—N1—C11	119.48 (19)
N1—C16—H16	110.1	C13—N1—C16	110.0 (2)
C17—C16—H16	110.1	C11—N1—C16	111.78 (17)
			
C10—C1—C2—C3	3.7 (12)	C15—C16—C17—C18	133.1 (10)
C12—C1—C2—C3	178.6 (9)	C22—C17—C18—C19	75.1 (2)
C1—C2—C3—C4	−4.1 (12)	C16—C17—C18—C19	−158.35 (18)
C2—C3—C4—C5	1.2 (13)	C22—C17—C18—C11	−160.27 (17)
C3—C4—C5—C10	1.9 (12)	C16—C17—C18—C11	−33.8 (2)
C3—C4—C5—C6	−179.0 (7)	N1—C11—C18—C19	160.73 (19)
C8—C7—C6—C5	1.0 (17)	C9—C11—C18—C19	29.7 (6)
C10—C5—C6—C7	−0.2 (12)	C12—C11—C18—C19	−86.0 (5)
C4—C5—C6—C7	−179.3 (9)	N1—C11—C18—C17	35.5 (2)
C6—C7—C8—C9	0(2)	C9—C11—C18—C17	−95.6 (5)
C7—C8—C9—C10	−2(2)	C12—C11—C18—C17	148.7 (4)
C7—C8—C9—C11	179.9 (12)	C17—C18—C19—O2	−1.1 (3)
N1—C11—C9—C8	−69.0 (17)	C11—C18—C19—O2	−120.9 (3)
C18—C11—C9—C8	53.7 (17)	C17—C18—C19—O3	−179.77 (18)
C12—C11—C9—C8	173.2 (16)	C11—C18—C19—O3	60.4 (2)
N1—C11—C9—C10	113.0 (8)	C16—C17—C22—O4	−42.8 (2)
C18—C11—C9—C10	−124.3 (8)	C18—C17—C22—O4	75.2 (2)
C12—C11—C9—C10	−4.7 (11)	C16—C17—C22—C26	80.7 (2)
C4—C5—C10—C1	−2.3 (11)	C18—C17—C22—C26	−161.32 (17)
C6—C5—C10—C1	178.5 (7)	O5—C23—C24—O6	−18.3 (2)
C4—C5—C10—C9	177.4 (10)	O4—C23—C24—O6	102.3 (2)
C6—C5—C10—C9	−1.8 (13)	O5—C23—C24—C25	−136.6 (2)
C2—C1—C10—C5	−0.6 (12)	O4—C23—C24—C25	−16.0 (3)
C12—C1—C10—C5	−176.6 (8)	O6—C24—C25—O8	163.24 (19)
C2—C1—C10—C9	179.7 (9)	C23—C24—C25—O8	−81.0 (2)
C12—C1—C10—C9	3.7 (11)	O6—C24—C25—C26	−81.9 (2)
C8—C9—C10—C5	3.0 (18)	C23—C24—C25—C26	33.9 (3)
C11—C9—C10—C5	−178.7 (7)	O4—C22—C26—O7	66.0 (2)
C8—C9—C10—C1	−177.2 (12)	C17—C22—C26—O7	−56.5 (2)
C11—C9—C10—C1	1.1 (13)	O4—C22—C26—C25	−48.7 (2)
C2—C1—C12—O1	−5.6 (19)	C17—C22—C26—C25	−171.18 (18)
C10—C1—C12—O1	169.8 (12)	O8—C25—C26—O7	−3.2 (3)
C2—C1—C12—C11	178.3 (8)	C24—C25—C26—O7	−120.3 (2)
C10—C1—C12—C11	−6.3 (9)	O8—C25—C26—C22	115.5 (2)
N1—C11—C12—O1	64.7 (12)	C24—C25—C26—C22	−1.6 (3)
C9—C11—C12—O1	−169.7 (12)	O2—C19—O3—C20	2.1 (4)
C18—C11—C12—O1	−44.9 (13)	C18—C19—O3—C20	−179.1 (3)
N1—C11—C12—C1	−119.0 (6)	C21—C20—O3—C19	83.4 (4)
C9—C11—C12—C1	6.6 (9)	O5—C23—O4—C22	81.7 (2)
C18—C11—C12—C1	131.5 (6)	C24—C23—O4—C22	−35.8 (3)
C10'—C1'—C2'—C3'	−4(3)	C26—C22—O4—C23	70.9 (2)
C12'—C1'—C2'—C3'	−178 (2)	C17—C22—O4—C23	−163.39 (17)
C1'—C2'—C3'—C4'	−8(3)	O4—C23—O5—C27	−125.5 (2)
C2'—C3'—C4'—C5'	14 (3)	C24—C23—O5—C27	−2.1 (2)
C3'—C4'—C5'—C6'	175 (2)	O6—C27—O5—C23	21.6 (2)
C3'—C4'—C5'—C10'	−8(3)	C28—C27—O5—C23	136.7 (2)
C8'—C7'—C6'—C5'	−1(4)	C29—C27—O5—C23	−98.4 (3)
C4'—C5'—C6'—C7'	180 (2)	C25—C24—O6—C27	156.06 (18)
C10'—C5'—C6'—C7'	2(3)	C23—C24—O6—C27	32.1 (2)
C6'—C7'—C8'—C9'	−3(5)	O5—C27—O6—C24	−33.8 (2)
C7'—C8'—C9'—C10'	6(5)	C28—C27—O6—C24	−149.8 (2)
C2'—C1'—C10'—C9'	−177 (2)	C29—C27—O6—C24	85.3 (2)
C12'—C1'—C10'—C9'	−2(3)	O8—C30—O7—C26	33.6 (3)
C2'—C1'—C10'—C5'	9(3)	C31—C30—O7—C26	148.4 (3)
C12'—C1'—C10'—C5'	−175.6 (18)	C32—C30—O7—C26	−86.6 (3)
C8'—C9'—C10'—C1'	−179 (3)	C22—C26—O7—C30	−138.0 (2)
C8'—C9'—C10'—C5'	−5(4)	C25—C26—O7—C30	−18.6 (3)
C4'—C5'—C10'—C1'	−3(3)	O7—C30—O8—C25	−35.8 (3)
C6'—C5'—C10'—C1'	174.8 (19)	C31—C30—O8—C25	−151.7 (3)
C4'—C5'—C10'—C9'	−177 (2)	C32—C30—O8—C25	83.2 (3)
C6'—C5'—C10'—C9'	1(3)	C24—C25—O8—C30	146.0 (2)
C10'—C1'—C12'—O1'	−176 (3)	C26—C25—O8—C30	23.9 (3)
C2'—C1'—C12'—O1'	−1(4)	C14—C13—N1—C11	145.8 (4)
N1—C13—C14—C15	−38.9 (8)	C14—C13—N1—C16	14.6 (5)
C13—C14—C15—C16	46.4 (11)	C9—C11—N1—C13	−24.6 (6)
C14'—C15'—C16—N1	−2(3)	C18—C11—N1—C13	−154.7 (3)
C14'—C15'—C16—C17	−125 (2)	C12—C11—N1—C13	90.5 (5)
C14'—C15'—C16—C15	−67 (18)	C9—C11—N1—C16	105.8 (5)
C14—C15—C16—C15'	79 (19)	C18—C11—N1—C16	−24.3 (2)
C14—C15—C16—N1	−39.1 (12)	C12—C11—N1—C16	−139.1 (4)
C14—C15—C16—C17	−154.6 (7)	C15'—C16—N1—C13	6(2)
C15'—C16—C17—C22	−96 (2)	C17—C16—N1—C13	138.8 (2)
N1—C16—C17—C22	140.62 (19)	C15—C16—N1—C13	15.1 (9)
C15—C16—C17—C22	−105.1 (10)	C15'—C16—N1—C11	−129 (2)
C15'—C16—C17—C18	143 (2)	C17—C16—N1—C11	3.6 (2)
N1—C16—C17—C18	18.9 (2)	C15—C16—N1—C11	−120.1 (9)

### Hydrogen-bond geometry (Å, °)

**Table:**

*D*—H···*A*	*D*—H	H···*A*	*D*···*A*	*D*—H···*A*
C4—H4···O8^i^	0.93	2.37	3.252 (9)	157
C26—H26···O1^ii^	0.98	2.38	3.211 (7)	143

Symmetry codes: (i) *x*−1, *y*−1, *z*; (ii) *x*, *y*+1, *z*.
